# The Successes and Failures in Our Daily Work1Read before the American Academy of Dental Science, Boston, February 1, 1905.

**Published:** 1905-11

**Authors:** Harry F. Hamilton

**Affiliations:** Boston, Mass.


### THE SUCCESSES AND FAILURES IN OUR DAILY WORK.[1]

BY DR. HARRY F. HAMILTON, BOSTON, MASS.

Mr. President, and Gentlemen of the Academy,—Instead of presenting a paper for your consideration this evening, I have

---

[1] Read before the American Academy of Dental Science, Boston, February 1, 1905.

thought it might be of more interest to take a few subjects, in which I had been working, and briefly present them for your consideration. While there is nothing perhaps distinctly novel, yet the successes and failures, the methods and results, of our daily work is always of interest and advantage to others.

### NAPKINS.

I wish to show first the napkins I use. We cannot control the laundry, and do not know what other articles are in the tub besides napkins, so that, while they look clean, I do not feel that they are so, and prefer not to use them a second time. After some search I found a heavy bleached cotton, rather stiff with dressing, which answers admirably, and after use is thrown away. It is the Anchor brand; one yard of it costs twelve and one-half cents and makes sixteen napkins; so you see the daily cost is small. Before being used I put them in the formalin sterilizer, so there is no possibility of odor. A pile of these new clean napkins in the drawer is a daily satisfaction, which I hope you will all try.

### RIBBON.

I next submit something, not as necessary, but which in many cases is useful. It is a very thin ribbon, without any extra thickness at the selvage, called lute string or taffeta ribbon, and which I use in cleaning teeth; and sometimes I can get patients to use it themselves, a few times a week with tooth-powder. It is not very strong, and in many places tears, but where it does go in will do its work quickly and well.

I pass around enough silk and napkins, so each gentleman may take one as a sample if he desires.

### NITRATE OF SILVER.

About twelve years ago I began using nitrate of silver, at first carefully, as I had some fear of its deep staining, but later I used it freely, and for ten years I have made it a rule to see that all the teeth back of the cuspids were given a good treatment of the saturated solution as soon as possible after eruption. I simply dry off the surfaces and put on the solution with a small swab, letting it stay a minute, during which time I push it with an explorer down into the sulci. I merely wish to give this as an invariable rule, carried out with great success for this length of time. The staining is only surface, and decay is generally prevented, and in cases where it does occur is greatly delayed. Teeth given this treatment need as careful watching as if untreated, for in a few cases it seemed that the nitrate did not go to the bottom, and decay went on, deep down, without showing on the surface. Our big operations give a certain satisfaction, but it is not to be compared to that of feeling that we have actually kept the teeth from decay.

CROWNS.

I have done much crown-work, my experience even going back to resetting a few old hickory pivots. I have followed all the fortunes, good and bad, of Bonwill and Richmond, and all their modifications, and I have arrived at the conclusion that there is only one crown, possessing all the qualifications of beauty, strength, and durability, and that is a porcelain crown on a banded platinum base,—the Richmond crown with platinum band and solid porcelain top.

There is one point I wish to bring out first, which I do not remember having seen in print, and that is the tolerance of the gums to platinum bands and bridges. The gums do not recede, but, on the contrary, embrace the band, and are perfect in color and cleanness. Gradually I have worked out a method which is simple and easy, and the result is quite perfect; but yet it takes much time, and I hope I shall be able to shorten it. I dare say you have all made similar crowns, but my method may be of interest.

I trim the root, as for a Richmond crown, only smaller, fit a copper band to the root, and, with that as a model, have a seamless platinum cap struck up, which I fit to the root. I then decide whether the pin is to be a part of the root or of the tooth. Generally I prefer to set a screw pin in the root. In this case, I put a hole in the cap, and solder a very thin platinum tube to it, through which the pin projects when set. The tooth is carved and baked on this base, and generally set with gutta-percha. It is equally valuable for bridge-work piers; and so easy to do that wonderfully good work can be done in the back of the mouth.

In the other case the pin is soldered to the cap, which it goes through, and is left projecting above it, to aid the porcelain's hold.

While these crowns may take a little more time to make, yet it is a straight operation; that is, each piece fits as it goes along, so when the crown is ready it goes perfectly into place, and there is no splitting, no decay of the root, but a crown absolutely strong, beautiful, and permanent as anything the dentist is called on to do.

INLAYS.

Two years ago I read a paper that Dr. Wassell, of Chicago, had delivered in Stockholm, the previous summer, telling his method of making inlays by the impression method. It seemed feasible, and later I chanced to see some of his work, which was superb. On my way to California I stayed over in Chicago, and saw much more of it; and it was a revelation. I saw mouths where careful examination was necessary to discover the inlays, and yet they comprised fillings in all sorts of places, and including large portions of the teeth. After seeing that beautiful exhibition of skill, I could not be satisfied with the inlay work which I had been doing, and which I had, indeed, been rather proud of, but I took steps to learn his methods, which are well described in the directions coming with Brewster's porcelains, and which, I presume, are familiar to you all.

The impression of the cavity is taken in cement, and the counter-die from it also in cement; the platinum matrix is swaged, and the inlay built to shape by trying in a plaster case of the teeth. With this method it is possible to build up corners even, and whole crowns, and have all the contours accurately reproduced, as in the original tooth. My first step on beginning was to install a young man in the laboratory to work up a knowledge of the methods. We have worked long and hard, and with many trials and discouragements, lightened by seemingly wonderful successes, until now it is comparatively easy for both, and we are looking toward the rewards.

You may recall, at an Academy meeting, a few years since, my showing the Jenkins apparatus, the first which we had seen. Before that I made some few inlays, with platinum matrix and Downie body, which, by the way, are excellent now; but when the elaborate Jenkins porcelains and furnace came, Dr. Hadley and I used it with much success, because we never ventured beyond the limitations of a low-fusing body. The inlays were quickly made, and little trouble arose from shrinkage. With the desire to build up the missing portions of the back teeth, new problems in shrinkage and repeated fittings arose, which could not be done in the mouth, and which in the laboratory need the greatest care to solve.

To better show the difficulties, I ought to say that although the man who has taken this in charge has shown much cleverness in his work, yet the result is that he can turn out only about fifteen satisfactory inlays a week; but as he now knows porcelain better, and has a helper for making the matrices, he will be able to furnish all we need.

This brings me to the point I wish to make, and which I have not seen made in inlay discussions, and that is, that the large inlays, involving more than one surface, can not be successfully made at the chair by a dentist with a large practice. We do not do our own artificial work; we do not make our own instruments; and yet this is infinitely more delicate, and, from my experience, I do not believe that any dentist, in the course of his daily work, can fit matrices, carefully dry his porcelain, and accurately bake it, with the pressure of coming patients hurrying him on. I have no doubt but that in the future some methods may be found to shorten and simplify this, which will modify this statement, but I believe the inlays, such as I show, should be made in a public or private laboratory.

I will pass around a few specimens, duplicates of actual cases that I have set. In these cases the maker of the inlays has not seen the tooth. I took the cement impression, and also one in impression material, gave them to him with the color, and he sent back the inlay.

As to inlays coming out, I regard that as a bugbear, and where they fail it is from the improper shape, or, more often from not keeping them absolutely dry for the hour needed while the cement is hardening. We never pay much attention to the shape of a cavity for a cement filling, and as inlays are at present made, they are nothing but cement fillings with nearly the whole of the cement replaced by porcelain.

At one time it looked as if inlays were to be permanently discredited, by the failures caused by dentists putting them in mouths before they had mastered the methods; but the successes have more than offset the failures, and little is heard now from the patients, to their discredit.

Before a discussion of these subjects, I would suggest that if any member would like to ask further information, I would be pleased to answer now.

QUESTIONS AND DISCUSSION.

*Dr. Piper.*—I would like to ask Dr. Hamilton about the platinum cap for the crown. Does he swage that in the office?

*Dr. Hamilton.*—No; I send it to the Paine Crown Company, who are expert in making them.

*Dr. Piper.*—I am glad to know that; I make the same thing on a Richmond crown. I would like to ask why you make your measurement in copper rather than in wire measurement?

*Dr. Hamilton.*—It gives the gum contour better.

*Dr. Clapp.*—Do you ever have any trouble with this thin edge of body running down over this band and cracking off after it has been in the mouth for some time?

*Dr. Hamilton.*—Very little. In one case it was on the inside, and I didn't mind it at all. If at any time it cracks off, I can heat it, take it off, roughen the platinum, and put more body on, with very little trouble.

*Dr. Baker.*—Give the method of setting with gutta-percha?

*Dr. Hamilton.*—I have used gutta-percha for some time by having it made in a big lump, and then with a rubber file having it filed down into a powder, and with oil of cajuput, a small amount, I would put it on a slab and warm it, and that would soften it up. About burning, I made a rule that if I could hold it in my fingers, the patient should be able to bear it on the gum.

*Dr. Cook.*—What form of gutta-percha do you use?

*Dr. Hamilton.*—Red base plate.

*Dr. Cook.*—Do you put anything with it?

*Dr. Hamilton.*—No, just as it comes.

*Dr. Merriam.*—I am somewhat in doubt whether I ought to speak on this matter of gutta-percha again or not, but this whole matter in relation to the setting of crowns over gutta-percha, the use of the essential oils for softening gutta-percha, and the setting of crowns by methods of impression, was brought before this Academy by a member long years ago, and, like many suggestions of the kind, it has never received recognition until brought back again from some other city. I will say that this matter of setting crowns in gutta-percha was presented also in New York at one of the meetings of the society there, and I presume it was recorded, although I am not sure.

*Dr. Werner.*—Many seeming innovations are only rehashed and reapplied methods. Take, for instance, our turbine engine, reinvented about thirteen years ago by a Swedish engineer. Hieron, in Alexandria, about the beginning of the Christian era, had it and used it.

Speaking of gutta-percha and the oil of cajuput and other essential oils, I remember Dr. Merriam speaking about it in the Odontological Society when he was a member there. He taught us that.

When I set a crown in gutta-percha I do not dance around in the way Dr. Hamilton speaks of, but let my patient do it. I simply have two or three finger cloths on my hands, and have an instrument carved out of wood on which I put my crown and warm it, and try that on my own hand, to see how well the patient can stand it. If I can stand it on my own hand, the patient can stand it very much better on a partially cocainized gum.

I have often thought that the crowns do crack if baked over this platinum. I also find difficulty with my platinum bands. They do not take very kindly to the gum. The gums that I have treated did not take kindly to bands of any kind. Seemingly they did take very kindly for three or four months, and sometimes for a year or two, but later those crowns look very bad.

I am not talking against inlays at all, for, however limited their usefulness, they have come to stay, but all of you have equipped yourselves with furnaces and complicated machineries, and the dental depot men will have much less interest in your buying teeth. There has been a great overdoing in inlays. In their proper place they are very good things, and should be studied by all of us.

*Dr. Allen.*—I want to ask Dr. Hamilton if he has ever used an iridio-platinum plate for bands. I find in my experience that the platinum bands made in this way cause trouble by binding and breaking off porcelain, and I use No. 30, as Dr. Hamilton has indicated, but I find that I can use No. 35 or 36 iridio-platinum, having it much thinner and safer, and with less danger of breaking.

*Dr. Hamilton.*—I have made some bands, but never a cap of iridio-platinum for crowns. I do not think it would be a good plan, as it has a way of cracking when stretched, and might develop an incipient crack which would open later.

*Dr. Allen.*—I have used them as I would make a Richmond cap, soldering platinum over the band instead of iridio-platinum. For the cap, on the edge of the band, I would use pure platinum.

*Dr. Merriam.*—I think that the soldering on platinum or iridio-platinum is apt to be treacherous. I find, in making the band for bicuspid crowns, we can make crowns entirely of iridio-platinum, and it appears to be strong, but after a time it peels off.

*Dr. Baker.*—I would like to ask Dr. Hamilton how he shapes his root?

*Dr. Hamilton.*—I have no particular way of shaping the root. Just with the disks and some good strong scalers.

*Dr. Werner.*—I would like to ask Dr. Hamilton how long he has used the swaging process in the laboratory?

*Dr. Hamilton.*—Since a year ago last October.

*Dr. Porter.*—Will you tell us in a few words just what you do in making an inlay?

*Dr. Hamilton.*—I open up the cavity with the rapid chisels, and do nearly all the excavating with the new Gem cavity wheels. These, if used under water, are not very painful. Then adjust a copper matrix, dry the cavity thoroughly, and polish well with common talcum powder (not the perfumed kind). Then fill with Fellowship cement, well over the edges. After it hardens, remove matrix and start impression by putting a piece of wood against a strong edge and striking the other end of the wood with a small hammer. This impression is set in plaster, face uppermost, and the die obtained by putting Ames's cement into it, after it has been well rubbed with talcum. This gives the working model, which is set in plaster, in the steel cup of the Brewster outfit.

The platinum matrix, one-thousandth of an inch thick, is adapted to the die by swaging and burnishing. I do not know that this gives a more perfect fit than if adapted in the cavity, but it is done in the laboratory, and does not take my time, which is the special plea for it that I am advocating this evening.

